# The Developmental Transcriptome of *Aedes albopictus*, a Major Worldwide Human Disease Vector

**DOI:** 10.1534/g3.119.401006

**Published:** 2020-01-21

**Authors:** Stephanie Gamez, Igor Antoshechkin, Stelia C. Mendez-Sanchez, Omar S. Akbari

**Affiliations:** * Section of Cell and Developmental Biology, University of California, San Diego, La Jolla, California,; † Division of Biology and Biological Engineering, California Institute of Technology, Pasadena, California, 91125,; ‡ Group for Research in Biochemistry and Microbiology (Grupo de Investigación en Bioquímica Y Microbiología-GIBIM), School of Chemistry, Universidad Industrial de Santander, Bucaramanga, Colombia, and; § Tata Institute for Genetics and Society-UCSD, La Jolla, California

**Keywords:** *Aedes albopictus*, transcriptome, development, Zika, dengue, *Aedes aegypti*, RNA-seq

## Abstract

*Aedes albopictus* mosquitoes are important vectors for a number of human pathogens including the Zika, dengue, and chikungunya viruses. Capable of displacing *Aedes aegypti* populations, this mosquito adapts to cooler environments which increases its geographical range and transmission potential. There are limited control strategies for *Aedes albopictus* mosquitoes which is likely attributed to the lack of comprehensive biological studies on this emerging vector. To fill this void, here using RNAseq we characterized *Aedes albopictus* mRNA expression profiles at 34 distinct time points throughout development providing the first high-resolution comprehensive view of the developmental transcriptome of this worldwide human disease vector. This enabled us to identify several patterns of shared gene expression among tissues as well as sex-specific expression patterns. To illuminate the similarities and differences with *Aedes aegypti*, a related human disease vector, we also performed a comparative analysis between the two developmental transcriptomes, identifying life stages where the two species exhibit similar and distinct gene expression patterns. These findings provide insights into the similarities and differences between *Aedes albopictus* and *Aedes aegypti* mosquito biology. In summary, the results generated from this study should form the basis for future investigations on the biology of *Aedes albopictus* and provide a gold mine resource for the development of transgene-based vector control strategies.

*Aedes albopictus* (*Ae. albopictus*), the Asian tiger mosquito, is a medically important invasive species whose habitat range has significantly increased over the past 20 years (Benedict *et al.* 2007). Originally from East Asia and the islands of the Pacific and Indian Ocean, this species is now found in all continents except for Antarctica (Bonizzoni *et al.* 2013). The rapid expansion of *Ae. albopictus* has been attributed to its ecological plasticity, strong competitive aptitude, feeding behavior, vector competence, its ability to enter diapause to escape unfavorable seasonal conditions, and lack of effective control strategies (Reynolds *et al.* 2012). An increase in habitat range imposes a greater risk of transmitting several mosquito-borne pathogens such as Zika, chikungunya, and dengue virus (Rezza 2012; Shragai *et al.* 2017). Even though this species is considered a less efficient dengue vector than *Aedes aegypti* (*Ae. aegypti*), the primary vector of dengue virus and a closely related mosquito species, it is responsible for several outbreaks of dengue and chikungunya virus (Vazeille *et al.* 2007; Ratsitorahina *et al.* 2008; Pagès *et al.* 2009).

Today both *Ae. albopictus* and *Ae. aegypti* are found in most Asian cities and in large parts of the Americas (Lambrechts *et al.* 2011). Both *Ae. albopictus* and *Ae. aegypti* feed on humans in daylight hours and rest indoors (Harrington *et al.* 2001; Valerio *et al.* 2010; Dzul-Manzanilla *et al.* 2017) and have similar larval niches, but their distributions depend on local environmental conditions (Kraemer *et al.* 2019). Interestingly, there are differences in dominance of mosquito vectors along urban-rural gradients. *Ae. albopictus* is often found in urban and rural environments, whereas *Ae. aegypti* tends to be an urban vector utilizing artificial containers (Tsuda *et al.* 2006; Kraemer *et al.* 2015). When both populations of mosquitoes are present in the same ecological niche *Ae. albopictus* mosquitoes tend to outcompete *Ae. aegypti* (O’meara *et al.* 1995; Beilhe *et al.* 2012). It is hypothesized that *Ae. albopictus* can do this because it is a superior larval competitor (Bagny Beilhe *et al.* 2013). In addition, *Ae. albopictus* is found to be ecologically plastic where it can survive in cooler environments than *Ae. aegypti*, thus facilitating its spread to unconventional environments (Kraemer *et al.* 2019). To help combat this emerging mosquito, we need a better understanding of its biology to enable the innovation of effective control strategies.

Previously, a comprehensive developmental transcriptome study of *Ae. aegypti* was performed and provided insight into the complexity of the basic biology of these mosquitoes (Akbari *et al.* 2013a; Matthews *et al.* 2018). This enormous dataset has provided the community with a foundation of data enabling the functional characterization of novel genes and germline promoters (Akbari *et al.* 2014b) which have subsequently been used to develop highly potent Cas9 endonuclease expressing strains (Li *et al.* 2017; Akbari *et al.* 2014b), in addition to gene drives (Li *et al.* 2019). While diapause has been studied extensively for *Ae. albopictus* because of its importance to its survival in different environments (Urbanski *et al.* 2010a, 2010b; Reynolds *et al.* 2012; Diniz *et al.* 2017), and the genome has been sequenced (Chen *et al.* 2015), there is currently no developmental transcriptome available for *Ae. albopictus*. Therefore to fill this void, here we provide a comprehensive analysis of the *Ae. albopictus* transcriptome throughout development which will provide the community with an invaluable resource to mine. In addition, this will provide a unique opportunity to perform comparative analysis and may even enable the discovery of novel genes and regulatory elements which may prove useful for innovating genetic control strategies.

## Materials and Methods

### Mosquito strain

Mosquitoes used for RNA extraction were from wildtype *Ae. albopictus* which originated from San Gabriel Valley, located in the Los Angeles County, CA. Mosquito eggs were collected from oviposition traps set by the San Gabriel Valley Mosquito Control district. Collected eggs were from sites were *Ae. albopictus* was known to circulate. These eggs were then hatched, checked for the characteristic stripe of *Ae. albopictus*, and reared for 10 generations before performing collection experiments. Mosquitoes were maintained in insectary facility with a relative humidity of 70–80%, maintained at 28°, and with a 12 hr/12 hr light/dark cycle. Larvae were fed with ground fish food (TetraMin Tropical Flakes, Tetra Werke, Melle, Germany) and sex separated as pupae. Adults were maintained and fed with an aqueous solution of 10% sucrose. Females were blood-fed 3-5 days after eclosion on anesthetized mice. All animals were treated according to the Guide for the Care and Use of Laboratory Animals as recommended by the National Institutes of Health.

### Total RNA isolation

In order to obtain a preliminary overview of the development of *Aedes albopictus*, one replicate of each sample was flash-frozen at specific time points, and total RNA was extracted using the Ambion mirVana mRNA isolation kit (Ambion/Applied Biosystems, Austin, TX). The number of tissues used per sample can be found in Table S1. The total RNA for a second testes replicate was extracted using the Qiagen RNeasy Mini kit (Qiagen, Germantown, MD). All sample collections were staged in the incubator at a relative humidity of 70–80%, 28° with a 12-hr/12-hr light cycle until the desired time point was reached. Samples were then immediately flash frozen. The adult non-blood fed (NBF) carcass was processed at 3 d after eclosion, and the adult male carcass and testes were processed at 4 d after eclosion. After extraction, RNA was treated with Ambion Turbo DNase (Ambion/Applied Biosystems, Austin, TX). RNA integrity was assessed using RNA 6000 Pico Kit for Bioanalyzer (Agilent Technologies #5067-1513).

### Illumina sequencing

RNA-seq libraries were constructed using NEBNext Ultra II RNA Library Prep Kit for Illumina (NEB #E7770) following the manufacturer’s instructions. Briefly, mRNA isolated from ∼1 μg of total RNA was fragmented to an average size of 200 nt by incubating at 94° for 15 min in first strand buffer, cDNA was synthesized using random primers and ProtoScript II Reverse Transcriptase followed by second strand synthesis using NEB Second Strand Synthesis Enzyme Mix. Resulting DNA fragments were end-repaired, dA tailed and ligated to NEBNext hairpin adaptors (NEB #E7335). After ligation, adaptors were converted to the ‘Y’ shape by treating with USER enzyme and DNA fragments were size selected using Agencourt AMPure XP beads (Beckman Coulter #A63880) to generate fragment sizes between 250 and 350 bp. Adaptor-ligated DNA was PCR amplified followed by AMPure XP bead clean up. Libraries were quantified with Qubit dsDNA HS Kit (ThermoFisher Scientific #Q32854) and the size distribution was confirmed with High Sensitivity DNA Kit for Bioanalyzer (Agilent Technologies #5067- 4626). Libraries were sequenced on Illumina HiSeq2500 in single read mode, with an approximately similar depth of 30 million reads per sample, and a read length of 50 nt following manufacturer’s instructions. Base calls were performed with RTA 1.18.64 followed by conversion to FASTQ with bcl2fastq 1.8.4.

### Poly(A+) read alignment and quantification

The *Ae. albopictus* reference genome assemblies and gene models were retrieved from NCBI (canu_80X_arrow2.2, GCA_001876365.2 for genome assembly and ftp://ftp.ncbi.nlm.nih.gov/genomes/all/GCF/001/876/365/GCF_001876365.2_canu_80X_arrow2.2/GCF_001876365.2_canu_80X_arrow2.2_genomic.gff.gz for gene models) (Table S2). Reads from RNA-seq libraries were aligned to the *Ae. albopictus* genome using STAR aligner (Dobin *et al.* 2013) with default parameters with the addition of ‘–outFilterType BySJout’ filtering option and ‘–sjdbGTFfile GCF_001876365.2_canu_80X_arrow2.2_genomic.gtf’ GTF file. Gene models were quantified with featureCounts (Liao *et al.* 2014) using ‘-t exon -g gene_id -M–fraction’ options. TPM (Transcripts Per Million) and FPKM (Fragments Per Kilobase Million) values were calculated from count data using Perl script addTpmFpkmToFeatureCounts.pl (File S1). For depiction of data, TPM was chosen because it considers the combined effects of sequencing depth and gene length for the read counts and is a commonly used metrics for cross sample comparisons. Quality assessment of the data included calculating Pearson correlations, hierarchical clustering (measure: Euclidean distance; clusters: average linkage), and principal component analyses (PCA) between samples.

### Use of DESeq2 for the exploration of data

To obtain insights into the types of genes upregulated in sex-specific samples, we performed a DESeq2 analysis in R using counts from libraries from each sex (Love *et al.* 2014). The count data were imported into the DESeq2 framework and analyzed with default parameters. Due to the single replicate (with the exception of the male testes) collection of samples in our study, we cannot accurately identify differential expression among samples. This will only allow us to explore our data by obtaining fold-changes and identifying potential genes that are upregulated. In our sex-specific analyses, we further sorted genes with fold changes >20x.

### Clustering and Gene Ontology (GO) analysis

TPM values produced by featureCounts for 47 RNA-seq libraries were clustered using Mfuzz R software package (Kumar and Futschik 2007). Mfuzz uses fuzzy c-means algorithm to perform soft clustering, which allows cluster overlap and has been demonstrated to perform favorably on gene expression data. The resulting clusters were analyzed for overrepresentation of GO terms using a hypergeometric test implemented using the GOstats R software package (Falcon and Gentleman 2007). Pfam domains for the *Ae. Albopictus* gene set were identified by running hmmscan (Finn *et al.* 2011) and associated GO terms were added using pfam2go mapping downloaded from the Gene Ontology Consortium (The Gene Ontology Consortium 2019). Hypergeometric tests were performed separately for biological process, molecular function, and cellular component ontologies. Only GO terms with a *p*-value < 0.05 were selected. Sample dendrograms and PCA plots were generated in R and plotted with ggdendro and ggplot2 packages (Kassambara 2015).

### Comparative analysis between Ae. albopictus and Ae. aegypti transcriptomes

Orthologous gene pairs between *Ae. albopictus* and *Ae. aegypti* were identified using the best reciprocal BLAST hit approach. Briefly, protein sets for *Ae. albopictus* and *Ae. aegypti* were downloaded from NCBI (ftp://ftp.ncbi.nlm.nih.gov/genomes/all/GCF/001/876/365/GCF_001876365.2_canu_80X_arrow2.2/GCF_001876365.2_canu_80X_arrow2.2_protein.faa.gz and ftp://ftp.ncbi.nlm.nih.gov/genomes/all/GCF/002/204/515/GCF_002204515.2_AaegL5.0/GCF_002204515.2_AaegL5.0_protein.faa.gz, respectively), blast databases for both sets were constructed with ncbi-blast v.2.7.1+ and blastp searches of each set against the other were performed with default parameters. Blast hits were parsed with a Perl script collectBlastHits.pl (File S2) and the best hit for each query sequence in the other species was identified and retained as an ortholog only if it identified the original query sequence as the best hit when searched in the other direction using a Perl script findBestReciprocalHitWithScore.pl (File S3). No explicit e-value cutoff was specified during searches. Protein IDs were then translated to gene IDs and multiple proteins per gene were collapsed to produce a nonredundant set of 10,696 orthologous gene pairs, which represents 54.51% and 27.62% of genes encoded by *Ae. aegypti* and *Ae. albopictus* genomes, respectively. The best reciprocal hit procedure required a strict one to one correspondence between *Ae. aegypti* and *Ae. albopictus* genes. Because of the extensive duplication known to exist in the current version of *Ae. albopictus* genome (predicted to encode 38,719 genes compared to 19,623 in *Ae. aegypti*), many genes produced ambiguous mapping and had to be eliminated from the list of orthologs. Nevertheless, the ortholog set identified here faithfully captures the general trends of the developmental transcriptome as illustrated by very similar sample clustering patterns based on expression values of orthologs alone and of the full gene set (Figure 2 and 3). To quantify expression values of orthologous genes, RNA-seq reads from the *Ae. aegypti* and *Ae. albopictus* samples were aligned to corresponding genomes using STAR (*i.e.*, *Ae. aegypti* reads were mapped to *Ae. aegypti* genome, *Ae. albopictus* reads were mapped to *Ae. albopictus* genome) with default parameters with the addition of ‘–outFilterType BySJout’ filtering option. Gene counts were extracted with featureCounts using ‘-t exon -g gene_id -M–fraction’ options and complete species-specific GTF files. Count data for orthologous gene pairs were parsed from full featureCount tables and used to identify the fold-change and base mean between the two species for each developmental stage using DESeq2. DESeq2 was run with default settings. MA plots for each sample pair were generated with ggplot2.

### Data availability

*Ae. Albopictus* mosquito line is available upon request. All sequencing data has been made publically available at NCBI Sequence Read Archive (SRA) under Accession number (BioProject ID (PRJNA563095)). Supplemental material available at figshare: https://doi.org/10.25387/g3.11653461.

## Results

### Ae. albopictus developmental transcriptome timepoints

To establish a comprehensive global view of gene expression dynamics throughout *Ae. albopictus* development, we performed Illumina RNA sequencing (RNA-Seq) on one replicate for each of the 47 unique samples representing 34 distinct stages of development (Figure S1A). These time points incorporated 31 whole animal and 16 tissue/carcass samples. For example, for embryogenesis 19 samples were collected; the first three time points, 0-1 hr, 0-4 hr, and 4-8 hr embryos, capture the maternal-zygotic transition, whereas 16 additional embryo samples were collected at 4 hr intervals until 72 hr to capture the duration of embryogenesis. Samples from four larval stages (instars 1-4) and sex-separated early and late male and female pupae were collected to capture the aquatic life cycle. Additionally, whole dissected ovaries and carcasses (whole female bodies lacking ovaries) from NBF females and from females at 12 hr, 24 hr, 36 hr, 48 hr, 60 hr, and 72 hr post-blood meal (PBM) were collected to examine the pre-vitellogenic “resting stage” through the completion of oogenesis. Adult male testes and carcasses (lacking testes) were collected at four days post eclosion to investigate male-specific germline and somatic gene expression.

To achieve single nucleotide resolution we extracted total RNA from each sample and sequenced entire transcriptomes using the Illumina HiSeq2500, and generated 1.56 billion 50nt reads corresponding to total sequence output of 78.19 GB with close to 95% of the reads aligning to the most contiguous and complete *Ae. albopictus* assembly available (assembly: canu_80X_arrow2.2, strain: C6/36, VectorBase) (Figure S1A; Tables S1, S2, S3). On average, 33,271,957 Illumina sequencing reads were obtained per sample/library (Table S4). A previous developmental transcriptome alignment using *Ae. aegypti samples* showed up to 93% of uniquely mapped reads (Akbari *et al.* 2013a). We anticipated similar results for *Ae. albopictus*, however, we found about 45% of reads uniquely mapped (Figure S1A; Table S3). This low percentage likely reflects the high duplication of the current *Ae. albopictus* genome assembly.

### Global transcriptome dynamics

**Figure 1:**
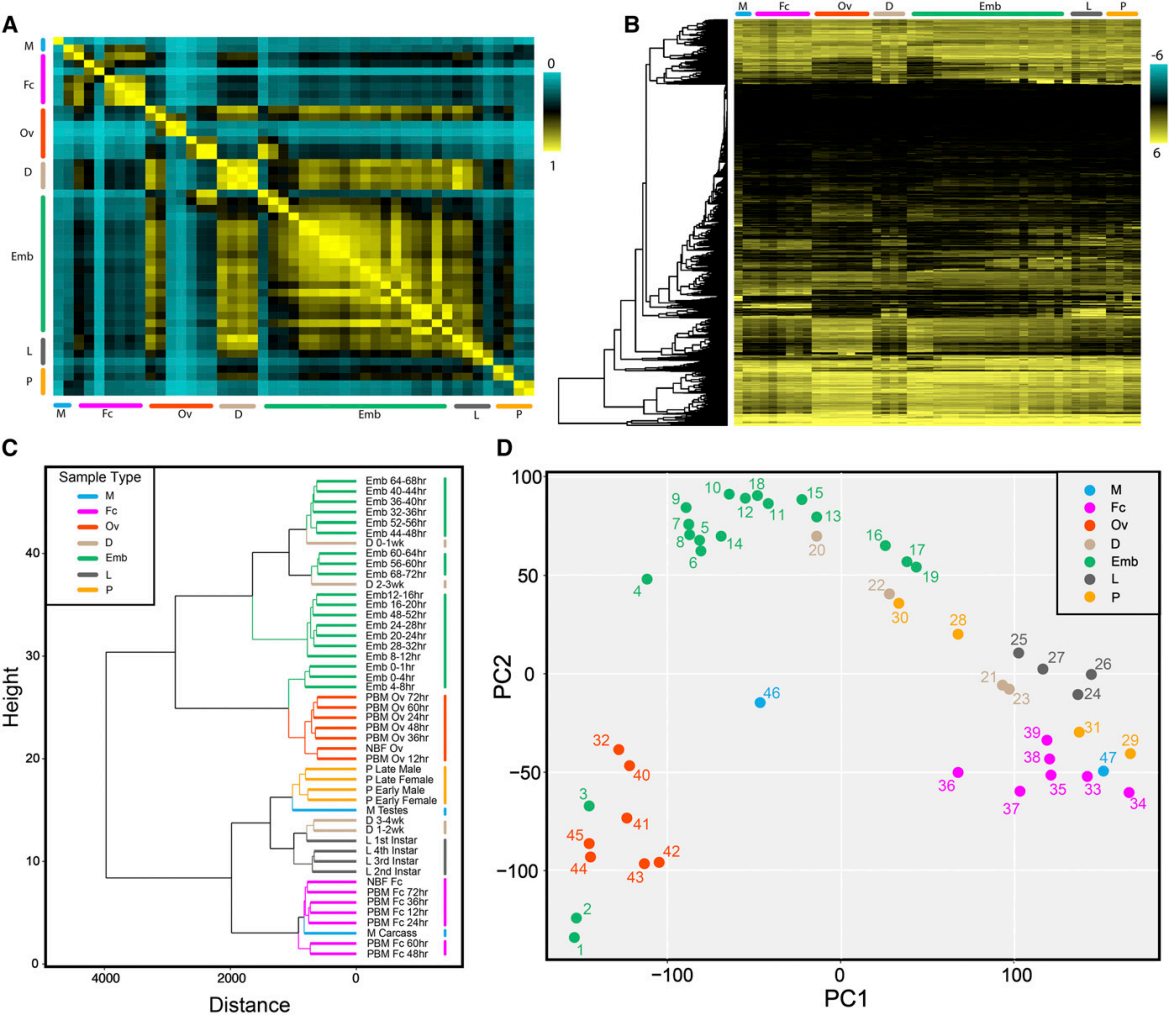
Global dynamics of gene expression. (A) Correlation matrix of all RNA seq timepoints for all known *Ae. albopictus* genes. (B) Hierarchical clustering heat map of albopictus genes across all developmental stages. FPKM values were log2(x+1) transformed and were scaled to plot the z-scores. (C) Dendrogram of *Ae. albopictus* samples clustering similar life stages closer together. Plot depicts the close relationship between all developmental samples. (D) PCA clustering of *Ae. albopictus* samples depicts clustering of life stages who show close similarity. PCA plot is in agreement with clustering dendrogram. Each point is labeled with the “Order” number they are assigned to from Table S1. For A-D, the second testes replicate was not shown. For A-D, the major developmental groups are indicated by color bars and are organized as follows: M (blue, male testes, male carcass), Fc (pink, NBF carcass, and multiple timepoints PBM: 12hr, 24hr, 36hr, 48hr, 60hr, and 72hr), Ov (orange, NBF ovaries, and multiple ovarian timepoints PBM: 12hr, 24hr, 36hr, 48hr, 60hr, and 72hr), D (tan, diapause at multiple timepoints: 0-1wk, 1-2wk, 2-3wk, and 3-4wk), Emb (embryo at multiple timepoints: 0-1 hr, 0-2 hr, 2-4 hr, 4-8 hr, 8-12 hr, 12-16 hr, 16-20 hr, 20-24 hr, 24-28 hr, 28-32 hr, 32-36 hr, 36-40 hr, 40-44 hr, 44-48 hr, 48-52 hr, 52-56 hr, 56-60 hr, 60-64 hr, 64-68 hr, and 68-72 hr embryos), L (gray, larvae 1^st^, 2^nd^, 3^rd^, and 4^th^ instar larvae stages), and P (yellow, pupae, early male and female, and late male and female pupae stages).

To capture the global dynamics of gene expression, we quantified the gene expression profiles across all developmental timepoints (Figure 1; Tables S4 and S5). Because this study is a developmental time course, the correlation between adjacent samples in the time course provide a reference to whether a sample is an outlier. According to Table S6, the correlation values between adjacent samples are extremely high as expected. The only exception was the male testes sample and for this we performed a second replicate to confirm our results. In general, the number of expressed genes (FPKM > 1) gradually increases through embryogenesis, reaching a peak at 68-72 hr (Figure S1B). This likely reflects the embryo developing and preparing for the next major developmental stage, the larval stage. In this stage, the number of genes expressed are fewer in 1^st^ larval instar, but then increases in the subsequent 2^nd^ to 4^th^ instars. After the larval stage, the animal undergoes metamorphosis into pupae where sexual dimorphism is apparent. During the early pupal stages, the number of genes increases suggesting transcripts involved in hormone production and initiation of adult formation are being expressed (Margam *et al.* 2006). In the adult stages the difference between the male and female germline is obvious with males expressing the highest number of genes. When females take a blood meal for egg production, the number of genes expressed in the ovary do not seem to vary notably, however when looking at their corresponding carcasses, varying levels across PBM females are observed suggesting dynamic gene expression in somatic tissues (Figure S1B). Interestingly, the tissue with the highest number of genes expressed corresponds to the male testes. In contrast, the lowest number of expressed genes correspond to the 24 hr PBM female carcasses. Analysis of pairwise correlations revealed that almost every developmental stage is most highly correlated with its adjacent stage and this is particularly evident during embryogenesis (Figure 1A). Notable exceptions to this trend occur in 24-36 hr PBM female carcass and 36-48 hr PBM ovaries, suggesting that these represent important points where physiological transitions occur in blood-fed females. Diapause samples (0 through 4 weeks) and NBF and 12 hr PBM ovaries are highly correlated with the mid-stages of embryogenesis suggesting similar genes are expressed in these samples. In the 0-1 hr embryo time point, we see similar gene expression with the 60- and 72- hr PBM ovaries likely reflective of maternally deposited transcripts. Samples with unique gene expression include the male germline, 24 hr PBM female carcass, 24 hr and 36 hr PBM ovaries, and late pupae (Figure 1A).

**Figure 2:**
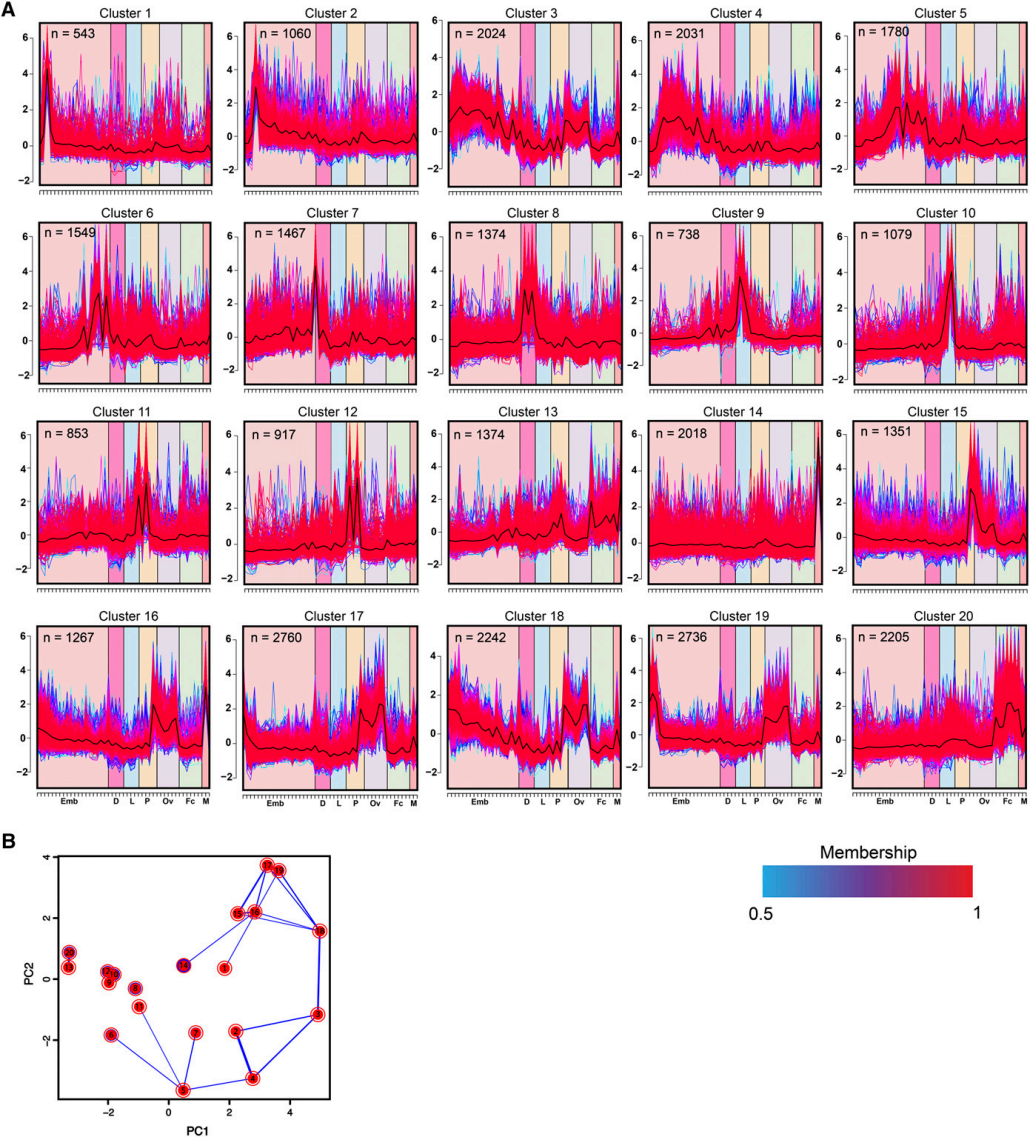
Soft clustering and principal component analysis on *Ae. albopictus* genes. (A) Twenty albopictus gene expression profile clusters were identified through soft clustering. Each gene is assigned a line color corresponding to its membership value, with red (1) indicating high association. The developmental groups are indicated by symbols on the x-axis. (B) Principal component analysis shows relationships between the 20 clusters, with thickness of the blue lines between any two clusters reflecting the fraction of genes that are shared. N, the number of genes in each cluster.

To further visualize the various patterns of gene expression and the relationships between the samples, hierarchical clustering and PCA analyses was performed (Figure 1B, C, D). Based on these analysis, embryo, PBM ovaries, pupae, larvae, and PBM female carcass samples tend to cluster closer together which is expected because their gene expression profiles are similar as these are developmentally related samples. Interestingly however, in Figure 1D, the male testes sample clusters away from all other samples, reflecting a distinguishing difference between this sample as compared to other samples sequenced. To observe patterns of co-regulated gene expression we used a soft clustering algorithm and identified 20 distinct patterns that included 543 to 2760 genes (Figure 2A). Each cluster in Figure 2A contains a set of *Ae. albopictus* genes that have an assigned membership value to indicate the degree of similarity to genes in that cluster (Table S7). The majority of these clustering patterns correspond to the developmental stages and transitions of the mosquito. For example, clusters 1 through 7 include genes that are associated with embryogenesis (Figure 2A). To investigate the functional associations of the genes in each cluster, we performed gene ontology (GO) analysis and focused on gene descriptions with *p*-values < 0.05 (Table S8). Here, we will focus on listing gene descriptions that are relevant to the mosquito’s developmental stage. Genes in clusters 1 through 7 are highly enriched in genes involving nucleic acid binding (*e.g.*, LOC109417994, LOC109422308 and LOC109424406), organic cyclic compound binding in the molecular function category. In the biological processes category, some highly enriched genes include macromolecule catabolic, metabolic, and biosynthetic processes (*e.g.*, LOC109410609, LOC109410731 and LOC109429162), DNA-templated transcription, and regulation of nitrogen compound metabolic processes (Table S8). These processes correspond to the necessities of the developing embryo as it transitions between stages with rapidly changing demands. Energy is supplied to the embryo through the breakdown of biomolecules. After embryogenesis, the deposited egg can enter diapause, a dormant state that allows the mosquito embryo to survive unfavorable conditions (Armbruster 2016). Cluster 8 includes gene expression required for diapause in dormant embryos. Here, several biological processes such as translation, amide biosynthetic, peptide biosynthetic and metabolic processes are enriched as well as molecular function terms associated with lipid transporter activity, lipid-A-disaccharide synthase activity, and ribonucleoside binding (Table S8). Genes enriched in this cluster include several lipases (to name a few: LOC109417138, LOC109401099, LOC109430899, and LOC109430905), several fatty acid hydroxylases (to name a few: LOC109400137, LOC109432075, and LOC109397180), and some proteases (LOC109406257, LOC109411917, and LOC109402104) (Table S8). This is likely due to the expression of genes that correspond to specific metabolic events associated with diapause to enable cold tolerance (Diniz *et al.* 2017). When an embryo hatches under favorable conditions it then enters the larval life-stage which is composed of four separate larval stages (1-4) before the pupal stage. In our clustering analysis, the larval stages correspond to clusters 9 and 10 (Figure 2A). In both of these clusters genes are enriched for serine-type peptidase activity, chitin binding, metallopeptidase activity, oxidoreductase activity, and ATPase activity under the molecular function category. Biological processes taking place include proteolysis, amino sugar metabolic, chitin metabolic, glucosamine-containing compound metabolic, and amino sugar metabolic processes (Table S8). The metabolic processes are likely involved in preparing the larva to acquire the energy reserves that will be used for egg development (Telang *et al.* 2006). Following the larval stage, the mosquito then enters the pupal stage, the final aquatic stage in the mosquito’s life cycle. Here, clusters 11, 12 and 15 correspond to the pupal stages and include terms in the molecular function category enriched for structural cuticular constituents, oxidoreductase, peptidase, and serine-type peptidase activity, which are likely involved in immunity and the hydrolysis of nutrients (Saboia-Vahia *et al.* 2013). Steroid biosynthetic and metabolic processes are also enriched which suggests hormones like ecdysteroids, which are crucial for metamorphosis, are preparing the pupa to molt into an adult mosquito (Margam *et al.* 2006).

Following the pupal stage, the aquatic life cycle ends with the animal undergoing metamorphosis into an adult. In males, the carcass and gonads have different terms enriched (clusters 13 for carcass and 14 for male testes). Cluster 13 include terms enriched for NADH dehydrogenase activity and oxidoreductase activity in the molecular function category. In the biological processes category, some terms enriched include several ribonucleotide and nucleotide metabolic processes (Table S8). In the male germline (cluster 14), some terms enriched in the molecular function category include microtubule motor activity, olfactory receptor activity, and neurotransmitter receptor activity. In the biological processes category, some terms enriched include spermatid development, microtubule based processes, sensory perception of chemical stimulus. Like *Anopheles gambiae* and *Ae. aegypti* mosquitoes, *Ae. albopictus* use chemosensation to activate their spermatozoa by modulating sperm activation and perhaps the orientation of spermatozoa (Pitts *et al.* 2014). Cluster 16 likely corresponds to the developing pupa and the developing germline of male and female pupae during the late pupal stages (Figure 2A, B). Here, there are several genes that peak in the late pupal samples, male testes, and late vitellogenic ovarian stage. Clusters 17, 18, and 19 correspond to ovarian development which consist of pre-vitellogenic (NBF ovaries) and vitellogenic (PBM ovaries) stages. In these ovarian developmental stages some genes that are enriched include cellular response to stimulus and Ras protein signal transduction which are important means of communication during the processes of oocyte and eggshell patterning (Dana *et al.* 2005). In addition, several metabolism processes that are crucial for the breakdown of organic molecules such as deoxyribonucleotides are crucial to support cell division (Telang *et al.* 2013). Finally, cluster 20 corresponds to genes that are expressed in PBM female carcasses. In the biological processes category, some terms are enriched for lipid metabolic processes and sensory perceptions and are both related to the processes required to support the future developing zygote in the ovaries. In the molecular function category, some relevant terms enriched include those with serine-type endopeptidase activity, serine hydrolase activity, and endopeptidase activity which are likely important for breaking down blood proteins (Bian *et al.* 2008).

### Sex-biased gene expression overview

Genes expressed in the germline of males and females are believed to play important roles in evolution, contributing to reproductive fitness, isolation, and speciation (Whittle and Extavour 2017). Thus it is important to study the role of sex-biased gonad genes in evolution. To gain some insight into the sex-biased differences in *Ae. albopictus*, we compared the transcriptomes of male and female samples (male testes and carcass, female NBF and PBM ovaries and carcasses, and pupal samples). In order to identify sex-biased genes, we performed a pairwise comparison for each sex-specific stage using a limited DESeq2 approach. For identifying biased genes in either the male or female germline, a total of seven pairwise comparisons were done including: Male testes *vs.* NBF ovaries; Male testes *vs.* 12 hr PBM ovaries; Male testes *vs.* 24 hr PBM ovaries; Male testes *vs.* 36 hr PBM ovaries; Male testes *vs.* 48 hr PBM ovaries; Male testes *vs.* 60 hr PBM ovaries; Male testes *vs.* 72 hr PBM ovaries. For identifying biased genes in either the male or female soma, a total of 8 pairwise comparisons were done including: Male carcass *vs.* NBF carcass; Male carcass *vs.* 12 hr PBM carcass; Male carcass *vs.* 24 hr PBM carcass; Male carcass *vs.* 36 hr PBM carcass; Male carcass *vs.* 48 hr PBM carcass; Male carcass *vs.* 60 hr PBM carcass; Male carcass *vs.* 72 hr PBM carcass; and Male pupae *vs.* Female pupae. In these pairwise analyses, identification of sex-biased genes were accomplished by looking at genes that were upregulated in females (indicated by negative log2FoldChange values) and males (indicated by positive log2FoldChange values). Because of the lack of replicates in our dataset, we cannot unambiguously assign significance values to the comparison results, which are intended to represent exploratory analysis in order to get an overall impression of sex-biased expression patterns. Further experimental characterizations will be required to confirm the expression dynamics of the identified genes. After generating a list of up and down-regulated genes in each sample, we decided to focus our discussion on a subset of genes, which display >20x fold overexpression in one of the sexes. Minimal expression can be expected from the opposite sex.

#### Female-biased genes in NBF and PBM states:

In our sex-biased comparison analysis, we found a combined total of 492 overexpressed genes that were upregulated in both the germline and somatic tissues of female mosquitoes and are listed in Table S9. Out of these genes, 128 have orthologs in *Ae. aegypti* while the rest seem to be specific to *Ae. albopictus*. This may be due to deficiencies in the annotation of the *Ae. albopictus* genome assembly or may represent loci that are specific to this species. Only 164 loci (out of 492) are uncharacterized. Additional work needs to be done to uncover the identity and function of these unknown loci that are highly expressed in the female ovaries. In NBF carcass samples, genes with >20x expression include 30kDa salivary gland allergens, trypsins, and several uncharacterized genes (Table S9).

Ingestion of vertebrate blood is essential for egg maturation by female mosquitoes. In order to gain insight into how a blood meal changes gene expression in female ovaries, we also analyzed PBM ovary and carcass samples (Table S9). A distinct pattern emerges when NBF females encounter a blood meal: genes expressed in NBF samples are downregulated in PBM samples and vice versa (Table S9). In PBM ovaries samples, vitelline genes become upregulated after a blood meal (beginning 12 hr PBM) and start to decrease at 36 hr PBM. Eventually their expression is downregulated to a point where almost no vitelline genes are sufficiently expressed (60 hr PBM). This may indicate a point where vitellogenesis halts and transitions to other oogenesis processes (Kokoza *et al.* 2001).

#### Male-biased genes:

In performing our global expression analysis, we found that the *Ae. albopictus* male testes sample clustered very distantly when compared to all other time point samples (Figure 1C, D). To ensure this was not due to an error in sample preparation or single replicate basis, we collected a second male testes replicate and performed a correlation analysis against all samples (Table S6). We find that both testes replicates are highly similar (*r* = 0.903) suggesting that the initial findings are supported. In our sex-bias comparison analysis, we found a combined total of 485 genes that were >20x upregulated in both types of male tissues (Table S10). Out of these only 220 are orthologous to *Ae. aegypti* genes. Among the genes with the highest upregulation in the male germline include several uncharacterized loci, cytosol aminopeptidases, tubulin chains, a couple kinases, and a 36.4 kD proline rich protein (Table S10). Other genes with relatively high prevalence in the testes include cilia- and flagella-associated proteins, cytochrome c oxidase subunits, dynein chains, and testis-specific serine/threonine protein kinases. It is likely that these genes are crucial for sperm development and management for male reproductive success. Among these exclusive genes we note an interesting observation. Two genes, LOC109414298 and LOC109407232 are found in different locations (Locations NW_017856468.1: 340,363-342,022 and NW_017856205.1: 8,069,187-8,070,779, respectively) and have 100% amino acid similarity to *Ae. aegypti*’s beta tubulin 2 protein sequence (Smith *et al.* 2007). While the nucleic acid sequence differed between *Ae. aegypti* beta tubulin 2 and *Ae. albopictus* LOC109414298 and LOC109407232, their amino acid sequences were identical. This suggests that *Ae. albopictus* contains at least two copies of the beta-tubulin 2 gene as paralogs. This finding is supported by a previous finding where twice as many seminal fluid proteins were identified in *Ae. albopictus* compared to *Ae. aegypti* (Degner *et al.* 2019). While we note there is duplication present in our species, we do not yet fully understand how two copies of a gene can affect mosquito biology of *Ae. albopictus*.

### Small RNA pathway protein dynamics

There are three major classes of regulatory small RNAs in animals that include: microRNAs (miRNAs), small interfering RNAs (siRNAs), and Piwi-interacting RNAs (piRNAs). Small RNAs are classified based on their size and interaction with Argonaute proteins. In addition, they are each involved in regulating particular processes in the mosquito. For example, miRNAs are shown to post-transcriptionally regulate transcript levels and the translational status of mRNA (Lucas *et al.* 2013). In mosquitoes, some miRNAs have been implicated in the regulation and function of blood digestion and ovarian development (Lucas *et al.* 2013). In contrast, the siRNA pathway is responsible for modulating arbovirus replication and can be responsible for transposable element silencing. Finally, the piRNA pathway is suggested to control the remobilization of transposable elements and may take part in antiviral immunity. In *Aedes* mosquitoes, there are seven PIWI proteins each with a seemingly distinct function (Miesen *et al.* 2015, 2016). To gain a global view of genes involved in small RNA processing we generated a heatmap to visualize their expression across development (Figure S2; Table S11). Increased expression of two piRNA genes, mael (AALF019672) and gstf (AALF023639), were apparent in NBF and PBM ovaries (Figure S2). Interestingly, twin (AALF018294), a CCR4 deadenylase, is highly expressed when the embryos undergo diapause. In *Drosophila*, this gene is shown to promote the decay of specific mRNAs in the early embryo (Rouget *et al.* 2010). In *Ae. albopictus*, it may serve as a mechanism to ensure diapause is transcriptionally arrested until there is an environmental signal that induces diapause termination. In contrast to all the stages, the male and female NBF and PBM germline contained piwi2 and piwi3 to be highly expressed (Figure S2). While there are several functions for the use of piRNAs in the insect germline, it is likely this observation suggests that the PIWI pathway is involved in silencing retrotransposons (Kalmykova *et al.* 2005; Wang and Elgin 2011). Piwi3 was found to be downregulated in male testes, later PBM ovaries, and in the early embryo (0-1 hr and 0-4 hr) (Figure S2). While there is still not much known about each particular PIWI protein, a study found that piwi3 may be associated with viral dissemination in mosquitoes (Wang *et al.* 2018).

### Comparison between Ae. albopictus and Ae. aegypti development transcriptomes

#### Transcriptome overview:

**Figure 3:**
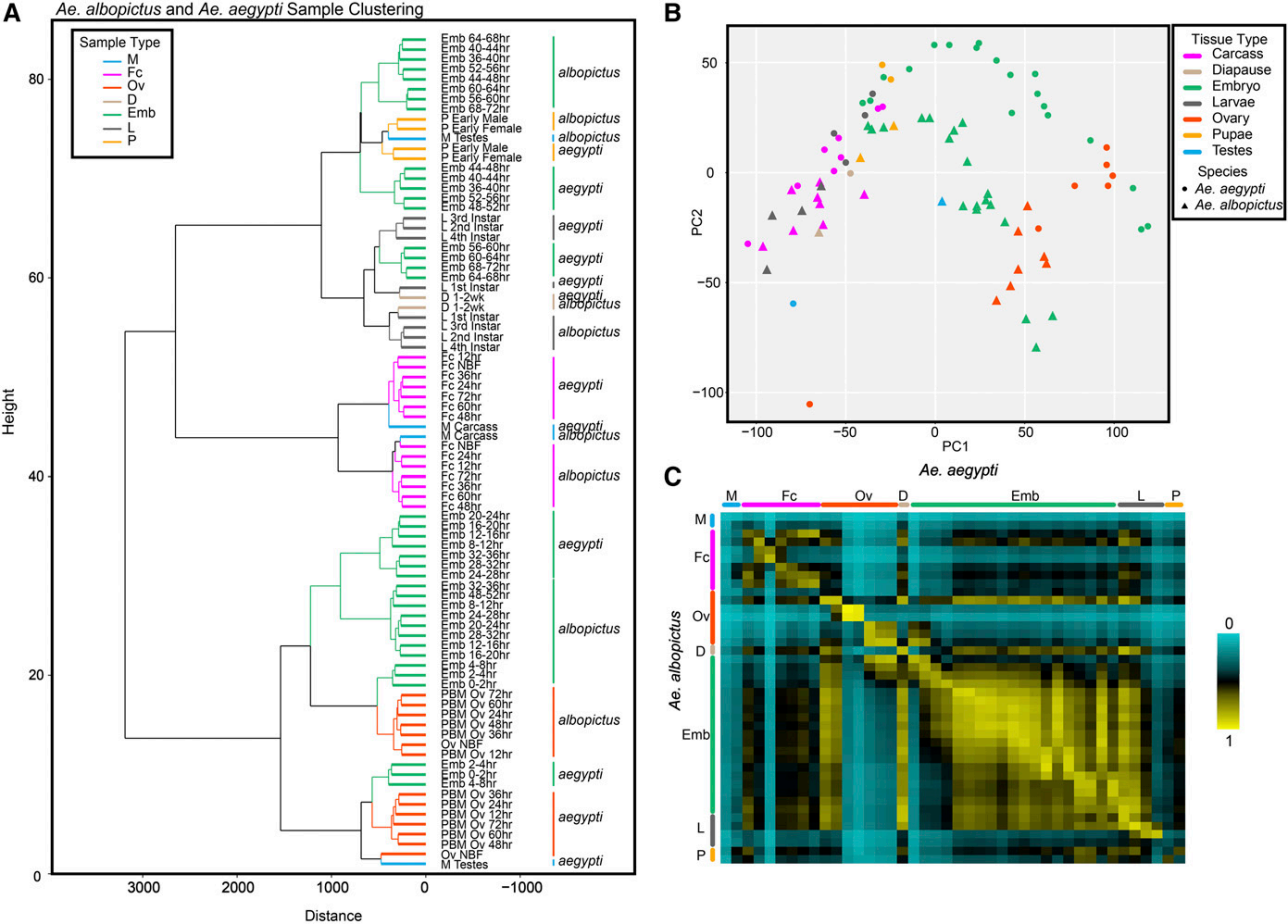
Orthology analysis of *Ae. aegypti* and *Ae. albopictus* samples across corresponding developmental time points. Orthologs were identified by best reciprocal BLAST hit approach and expression values of orthologous genes were determined by aligning RNA-seq reads from the *Ae. aegypti* and *Ae. albopictus* samples to corresponding genomes using STAR (*e.g.*, *Ae. aegypti* reads were aligned to *Ae. aegypti* genome) and quantifying them with featureCounts (Materials and Methods). Species-specific expression values of orthologous genes were used for clustering and PCA analyses. (A) Dendrogram and (B) principal component analysis (PCA) on similar life stage sample types in both species. Both clustering analyses agree with each other indicating the close relationships of similar genes among each developmental time point between species. Interestingly, the *Ae. albopictus* male testes sample clusters distantly from *Ae. aegypti* testes which may indicate a significant difference between the two species. (C) Heat map of calculated Pearson correlations on samples between species. *Ae. albopictus* samples are represented on the vertical axis of the heat map while *Ae. aegypti* samples are represented on the horizontal axis. Life stages are indicated by the similar colored bars for both species.

We next sought to determine the similarities and differences between the developmental transcriptomes of *Ae. aegypti* and *Ae. albopictus* mosquitoes. To establish orthologs, we performed BLAST searches on the proteomes of both species and identified best reciprocal hits ensuring one-to-one relationship between genes in the two species resulting in 10,696 orthologous pairs representing 54.51% and 27.63% of genes encoded by *Ae. aegypti* and *Ae. albopictus* genomes, respectively (Table S12 for best reciprocal hits). For BLAST searchers, no explicit e-value cutoff was specified during searches, but only 8 of 11,687 identified orthologous protein pairs (0.068%) had blastp e-value above 0.001 (0.37 the highest). 11,666 of orthologs (99.820%) had e-value below 1e^-10^; 10,353 (88.586%) had e-values below 1e^-100^; and 8,033 (68.734%) had e-values of 0 (Figure S3). Using previously published *Ae. aegypti* developmental transcriptome data (Akbari *et al.* 2013a), we conducted a set of analyses comparing gene expression levels between the two species’ developmental stages with the aim to gain insight into possible differences in their biology. To determine expression values of orthologous gene pairs, RNA-seq reads from the *Ae. aegypti* and *Ae. albopictus* samples were aligned to corresponding genomes using STAR (*i.e.*, *Ae. aegypti* reads were mapped to *Ae. aegypti* genome, *Ae. albopictus* were mapped to *Ae. albopictus* genome). The reads were quantified with featureCounts using species-specific GTFs defining orthologous gene models. On average, 57.6% (range: 23.3–72.9%) and 34.6% (range: 29.1–39.4%) of RNA-seq reads from *Ae. aegypti* and *Ae. albopictus* datasets were mapped to orthologs, respectively (Table S13). Sample clustering and PCA analysis using expression values of orthologous genes revealed that the majority of the corresponding sample timepoints and tissues between both species display similar overall expression patterns and are found adjacent to each other (Figure 3A, 3B). A notable exception is the testes sample pair, which cluster far apart presumably reflecting considerably different gene expression programs. Finally, we calculated Pearson correlations between TPM values of the corresponding samples and then plotted them on a heatmap to confirm similarities between embryonic samples (Figure 3C, Figure S4, Table S14). Embryonic samples between species have higher correlations indicating that the genes involved in this developmental stage are very similar or shared. This is also true for all other tissues/time points, however, with the exception of the male testes, consistent with the results of clustering analysis (Figure 3C; Figure S4; Table S14).

We next performed DESeq2 analysis between *Ae. albopictus* and *Ae. aegypti* samples at each developmental time point to gain some insight into the differences in expression of orthologous genes (Table S15). As with the sex-bias analysis, these comparisons are exploratory in nature due to the lack of replicates. They are aimed at high-level overview of orthologous gene expression in the two species. In the lack of replicates, few genes reach significant adjusted p-values due to the likely overestimation of dispersion of count values by the DESeq2 algorithm making it impossible to draw definitive conclusions about genes with poor p-values. The genes that satisfy the significance criterion however may represent good candidates for truly differentially expressed genes and may provide a foundation for more detailed analyses in the future. Here we will focus on five samples (NBF ovaries, female pupae, 4^th^ instar larvae, male carcass, and male testes) that displayed very low pairwise correlations (*r* < 0.50) between species (Figure S4, Table S14) and genes that display adjusted *p*-values < 0.1 in DESeq2 tests (Table S15, Figures S5-S8). Raw data tables for all DESeq2 *Ae. albopictus-Ae. aegypti* tissue comparisons are found in Tables S16-S21.

When compared to *Ae. albopictus*, the *Ae. aegypti* female germline contains several upregulated genes. They include genes related to mitochondria and the production of energy (59 terms) (Table S15). GO terms enriched in this sample include ATP synthesis coupled proton transport, electron transfer activity, mitochondrial electron transport, and mitochondrial respiratory chain complex. In addition, several genes with DNA binding, RNA polymerase, protein binding, transcription, and translation were enriched. In *Ae. albopictus*, relatively fewer genes are upregulated in the female germline. Genes enriched include those associated with translation, ATP binding, DNA binding, and metabolic process . In the female pupal stage, Ae. aegypti contains genes enriched for terms that include chitin binding, chitin metabolic processes, serine-type endopeptidase activity, and many uncharacterized genes. Interestingly, *Ae. albopictus* contains genes enriched for odorant and protein binding and other terms. Compared to *Ae. albopictus*, *Ae. aegypti*’s 4^th^ larval instar program contains genes enriched for oxidoreductase activity, proteolysis, chitin binding, and steroid biosynthetic processes. In *Ae. albopictus*, upregulated genes correspond to translation, protein secretion, metabolic processes, oxidation-reduction, and metallopeptidase activity (Table S15). Metallopeptidases are a class of endopeptidases that are hypothesized to function in immunity and development(Vishnuvardhan *et al.* 2013).

In male carcasses, *Ae. aegypti* contains terms enriched for some metabolic processes, chitin binding, hydrolase activity and proteolysis. In *Ae. albopictus*, upregulated genes correspond to terms enriched for ATP binding, protein binding, and proteolysis. Based on our initial analyses of the *Ae. albopictus* samples, the male testes sample depicted major differences when compared to all stages (Figure 1). It was highly uncorrelated to other stages within this species and displayed a uniqueness that needed to be further investigated. When the testes sample was compared to *Ae. aegypti*, we found that more genes were upregulated in *Ae. albopictus* testes as compared to *Ae. aegypti* testes (172 *vs.* 97 genes, respectively) (Table S15). The majority of these orthologous genes in *Ae. albopictus* (n = 69) are uncharacterized and their function remains unknown (Table S15). It would be interesting to further explore how these unknown genes affect male germline development and fertility. Several annotated genes correspond to flagellar structure including microtubules, dynein, ciliar components, and proteins associated with mitochondrial derivatives (Table S15). Other genes include testis-specific protein kinases, histone genes, and several genes involved in DNA-binding transcription factor activity, presumably to regulate transcription in spermatogenesis. Several serine/threonine-protein kinases are expressed and are involved in the control of many physiological processes, like flagellar motility and muscle contraction (Cohen 1997). Cytosol aminopeptidase was also upregulated in *Ae. albopictus*. This gene is among one of the top highly expressed male-specifc genes in our *Ae. albopictus* testes analyses, with multiple copies present, and is known to be one of the most abundant sperm proteins in *Ae. aegypti* seminal fluid (Degner *et al.* 2019). In *Ae. aegypti* testes, we found fewer upregulated genes that include odorant binding proteins, carboxypeptidases, trypsins, and hormone-related genes (Table S15).

## Discussion

*Ae. aegypti* and *Ae. albopictus* are medically important mosquitoes with the capacity to transmit a variety of human pathogens. Taking into account that these mosquitoes are vectors of dengue, Zika and chikungunya viruses, there is a potential risk of increasing the incidence of these diseases. Although *Ae. aegypti* is considered as the primary vector for these viruses, *Ae. albopictus* is emerging as another important vector (Reiter *et al.* 2006). In order to contribute to the knowledge of the biological development of *Ae. albopictus*, we analyzed the whole transcriptome at different developmental stages of the life cycle. Our work will provide others with the basic foundation for their genomic studies. The observations presented here should reflect a comprehensive snapshot of the *Ae. albopictus* developmental transcriptome, an accomplishment not yet undertaken in the field–until now. Our results provide confirmation for up to 95% (36,347/38,261) of previously annotated AALF genes.

The mosquito’s life cycle can be divided into four major phases: the maternal to zygotic transition (ovary to embryos 0-8 hr); embryogenesis (from 8hr to 72 hr), diapause (0-1wk and 2-3wks); larvae (1-4 instars) to pupae (early and late); and adults (male and female). These crucial transitions of the mosquito life cycle share many genes whose expression shows little difference between each time point. Our cluster analysis and characterization of developmental and sex-biased genes identified a number of patterns of co-regulated gene expression. We see sex-biased expression of a number of genes in the mosquito male and female germline that gives us insights into the reproductive biology of *Ae. albopictus*. Identification of loci involved in the blood meal program of the ovaries will be of interest to understand the regulation of ovarian development. In this study, we were able to depict the dynamics of genes involved in small RNA production (siRNAs, miRNAs, and piRNAs) across all development. Small RNAs in mosquitoes are known to partake in many important roles in cell development, response to stress, infection, and the silencing of transposable elements (Lucas *et al.* 2013). While we did not characterize small RNAs, our analysis on genes involved in small RNA production gives us insights into the roles of the small RNA pathways in *Ae. albopictus*. It would be interesting to investigate the small RNA profiles as it pertains to our results.

In our transcriptomic analysis of mosquito tissues, we discovered that the male testes showed a distinct gene expression profile that differentiated it from other tissues. Upon closer inspection, we identified 485 male-biased genes that were expressed in male testes, carcass, and male pupae. Among this list, the highest expressing genes corresponded to several uncharacterized loci, cytosol aminopeptidases, and tubulins. It would be interesting to see what functions and processes the uncharacterized genes are involved in. It is likely they may be involved in spermatogenesis, seminal fluid production, or a mating induced response. Perhaps these highly expressed genes have important roles in spermatogenesis and in the management and production of seminal fluid proteins to enable the reproductive success of *Ae. albopictus* males. The highly divergent gene expression in the testes may suggest male-specific genes in this species are evolving rapidly (Swanson and Vacquier 2002). *Ae. albopictus* males exhibit an interesting mating strategy that involves male mosquitoes mating with multiple females in succession without regard to the availability of sperm in their testes (Oliva *et al.* 2013). Another strategy involves transferring male accessory gland secretions with sperm into the female to prevent further insemination. This is very similar to the mating plug seen in *Anopheline* species (Giglioli 1964). In the wild, *Ae. albopictus* mosquitoes are shown to displace *Ae. aegypti* populations in areas where they co-occur (Muzari *et al.* 2019). Currently, it is hypothesized that *Ae. albopictus* is able to do this because it is a superior larval competitor, however, a recent study suggested that competitive displacement is due to *Ae. albopictus* males mating with *Ae. aegypti* females resulting in female mating refractoriness (Tripet *et al.* 2011). This mating interference is known as ‘satyrization’ and has been suggested to be a form of adaptation favoring the invasive success of *Ae. albopictus* (Lounibos and Kramer 2016). In our search to understand what are the highly expressed genes in male testes, we found at least one beta-tubulin-2 gene that was duplicated. This is not surprising considering the *Ae. albopictus* genome is highly duplicated, however, it is unclear to what extent a duplication of male-biased genes can contribute to the male reproductive success of these mosquitoes. It is possible that in this species, the testes-biased genes can exhibit rapid evolution contrary to *Ae. aegypti* which experiences decelerated rates of evolution in the testes (Whittle and Extavour 2017).

The comparative *Aedes* developmental transcriptomics approach enabled us to obtain insights into the similarities and differences in developmental life stages between the two species. For example, when comparing the correlations of corresponding samples between species, the male testes has the lowest similarity and indicates an interesting difference in male germline biology in *Ae. albopictus* mosquitoes. The most similar sample/tissue between species was the 24 hr PBM ovaries. This similarity may suggest a conservation of genes involved in oogenesis at that time point. A DESeq2 analysis allowed for the exploration of our data and gave us insights into what kinds of genes were coregulated across developmental stages. Additional replicates will be needed to identify with accuracy and confidence the loci with differential expression. Overall, our results provide insight into the overall differences between these two species and list potential genes that may be involved.

In addition to providing a tool for basic molecular research on *Ae. albopictus*, the developmental transcriptome will facilitate the development of transgenesis-based control of vector populations. Regulatory elements that direct expression of transgenes in germline specific tissues will be useful for the development of gene drive mechanisms for spreading a desired trait in a mosquito population (Sieglaff *et al.* 2009; Akbari *et al.* 2014b). In *Ae. aegypti*, several regulatory elements able to drive gene expression in a tissue- and temporal- specific manner have been identified through extensive study (Akbari *et al.* 2013a) and transgenesis (Coates *et al.* 1999; Kokoza *et al.* 2000; Moreira *et al.* 2000; Smith *et al.* 2007). Future functional characterization of uncharacterized genes and regulatory elements may lead to the development of innovative genetic population control technologies such as precision guided sterile males (Kandul *et al.* 2019b), and gene drive systems (Akbari *et al.* 2013b, 2014a; Champer *et al.* 2016; Buchman *et al.* 2018b, 2018a; Kandul *et al.* 2019a,b; Li *et al.* 2019) which can be linked to anti-pathogen effectors (Buchman *et al.* 2019a, 2019b) potentially providing paradigm-shifting technologies to control this worldwide human disease vector. Overall, our results provide a comprehensive snapshot of gene expression dynamics in the development of *Ae. albopictus* mosquitoes. The comparative analysis performed between *Ae. albopictus* and *Ae. aegypti* will be helpful in facilitating future comparative biological studies to understand the molecular basis of their differences.
